# Postictal Psychosis in Epilepsy: A Clinicogenetic Study

**DOI:** 10.1002/ana.26174

**Published:** 2021-08-03

**Authors:** Vera Braatz, Helena Martins Custodio, Costin Leu, Luigi Agrò, Baihan Wang, Stella Calafato, Genevieve Rayner, Michael G. Doyle, Christian Hengsbach, Francesca Bisulli, Yvonne G. Weber, Antonio Gambardella, Norman Delanty, Gianpiero Cavalleri, Jacqueline Foong, Ingrid E. Scheffer, Samuel F. Berkovic, Elvira Bramon, Simona Balestrini, Sanjay M. Sisodiya

**Affiliations:** ^1^ Department of Neurology Neurological and Neurosurgical Clinic of Joinville Joinville Brazil; ^2^ Department of Clinical and Experimental Epilepsy UCL Queen Square Institute of Neurology London UK; ^3^ Chalfont Centre for Epilepsy Buckinghamshire UK; ^4^ Genomic Medicine Institute, Lerner Research Institute Cleveland Clinic Cleveland OH; ^5^ Stanley Center of Psychiatric Research Broad Institute of Harvard and Massachusetts Institute of Technology Cambridge MA; ^6^ Mental Health Neuroscience Research Department Division of Psychiatry, University College London London UK; ^7^ Melbourne School of Psychological Sciences University of Melbourne Parkville Victoria Australia; ^8^ Department of Clinical Neuropsychology Austin Health Melbourne Victoria Australia; ^9^ Epilepsy Programme, Department of Neurology Beaumont Hospital Dublin Ireland; ^10^ FutureNeuro Research Centre Royal College of Surgeons in Ireland Dublin Ireland; ^11^ Department of Neurology and Epileptology, Hertie Institute for Clinical Brain Research University of Tübingen Tübingen Germany; ^12^ IRCCS Istituto delle Scienze Neurologiche di Bologna, Full Member of the ERN EpiCARE, delle Scienze Neurologiche di Bologna (EpiCARE reference center) Bologna Italy; ^13^ Dipartimento di Scienze Biomediche e Neuromotorie Università di Bologna Bologna Italy; ^14^ Department of Epileptology and Neurology University of Aachen Aachen Germany; ^15^ Department of Medical and Surgical Sciences Institute of Neurology, University Magna Græcia Catanzaro Italy; ^16^ School of Pharmacy and Biomolecular Sciences Royal College of Surgeons in Ireland Dublin Ireland; ^17^ Department of Molecular and Cellular Therapeutics and FutureNeuro Research Centre Royal College of Surgeons in Ireland Dublin Ireland; ^18^ University of Melbourne, Austin Health, and Royal Children's Hospital Florey Institute and Murdoch Children's Research Institute Melbourne Victoria Australia; ^19^ Epilepsy Research Centre, Department of Medicine, Austin Health University of Melbourne Melbourne Victoria Australia; ^20^ Institute of Psychiatry, Psychology, & Neuroscience at King's College London London UK; ^21^ Institute of Cognitive Neuroscience University College London London UK; ^22^ Neuroscience Department Children's Hospital ‘Anna Meyer’‐University of Florence Florence Italy

## Abstract

**Objective:**

Psychoses affecting people with epilepsy increase disease burden and diminish quality of life. We characterized postictal psychosis, which comprises about one quarter of epilepsy‐related psychoses, and has unknown causation.

**Methods:**

We conducted a case–control cohort study including patients diagnosed with postictal psychosis, confirmed by psychiatric assessment, with available data regarding epilepsy, treatment, psychiatric history, psychosis profile, and outcomes. After screening 3,288 epilepsy patients, we identified 83 with psychosis; 49 had postictal psychosis. Controls were 98 adults, matched by age and epilepsy type, with no history of psychosis. Logistic regression was used to investigate clinical factors associated with postictal psychosis; univariate associations with a *p* value < 0.20 were used to build a multivariate model. Polygenic risk scores for schizophrenia were calculated.

**Results:**

Cases were more likely to have seizure clustering (odds ratio [OR] = 7.59, *p* < 0.001), seizures with a recollected aura (OR = 2.49, *p =* 0.013), and a family history of psychiatric disease (OR = 5.17, *p* = 0.022). Cases showed predominance of right temporal epileptiform discharges (OR = 4.87, *p* = 0.007). There was no difference in epilepsy duration, neuroimaging findings, or antiseizure treatment between cases and controls. Polygenic risk scores for schizophrenia in an extended cohort of postictal psychosis cases (n = 58) were significantly higher than in 1,366 epilepsy controls (*R* ^2^ = 3%, *p* = 6 × 10^−3^), but not significantly different from 945 independent patients with schizophrenia (*R* ^2^ = 0.1%*, p* = 0.775).

**Interpretation:**

Postictal psychosis occurs under particular circumstances in people with epilepsy with a heightened genetic predisposition to schizophrenia, illustrating how disease biology (seizures) and trait susceptibility (schizophrenia) may interact to produce particular outcomes (postictal psychosis) in a common disease. ANN NEUROL 2021;90:464–476

Psychiatric disorders are common in epilepsy, affecting up to 50% of patients^1^; prevalence is higher in focal epilepsies, particularly those involving the temporal lobe, than in idiopathic generalized epilepsies.^2^ The broad psychiatric spectrum in epilepsy encompasses affective disorders (with depression the most common), anxiety disorders, attention‐deficit/hyperactivity disorder, and psychoses.^3^, ^4^ The psychoses of epilepsy comprise interictal psychosis of epilepsy, unrelated to acute seizures; postictal psychosis (PIP), occurring after a lucid interval of up to 48 hours following seizures; and antiseizure medication (ASM)‐induced psychotic disorder, when the psychotic symptoms develop during or soon after exposure to ASM or during or soon after its withdrawal.^5^, ^6^, ^7^

Psychotic disorders affect about 6% of people with epilepsy; there is an almost 8‐fold increased risk of psychosis in people with epilepsy compared to the general population.^1^ In a Danish population‐based study, the incidence of schizophrenia (SCZ)‐like psychosis was 3 times higher in individuals with epilepsy than in the general population.^8^ The prevalence of PIP in epilepsy overall is estimated at 2%, rising to 7% in patients with temporal lobe epilepsy.^1^

The cause of PIP is unknown. The suggestion of a common pathophysiological mechanism or of a shared genetic susceptibility to both epilepsy and psychosis has emerged from previous studies.^9^, ^10^ Better understanding its clinical features would help its proper recognition and determination of its causation, both crucial for the best treatment. The aim of this study was to identify clinical and genetic features associated with PIP.

## Patients and Methods

**FIGURE 1:**
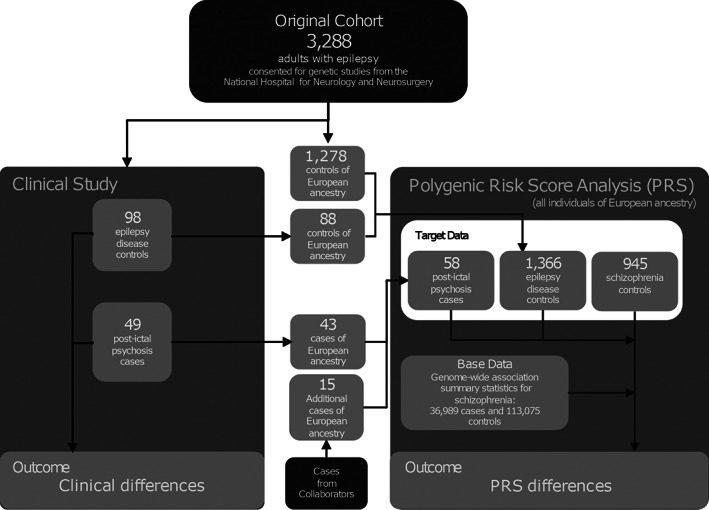
Flow chart of the study design.

This study was approved by the relevant institutional ethics committee at each center. All participants provided written informed consent. The study design is illustrated in Figure 1.

### Study Cohorts

We first studied adults with epilepsy from the National Hospital for Neurology and Neurosurgery, London, UK (NHNN). The inclusion criteria were (1) a diagnosis of epilepsy; (2) onset of delusions, hallucinations, or disorganized thinking and behavior within 1 week after a lucid interval following a seizure (or a seizure cluster); (3) impairment of social or occupational function; (4) duration ranging from 1 day up to 3 months; and (5) confirmation of the diagnosis by a psychiatrist or neuropsychiatrist. The exclusion criteria were (1) psychosis as part of ictal semiology or (2) psychotic symptoms/disorder preceding epilepsy. All the above criteria were confirmed by detailed review of the clinical records.

To permit genetic analysis, NHNN cases were selected from 3,288 patients included in a broad genetic study of epilepsy; records for 83 with any history of psychosis were reviewed in detail, and 49 who fulfilled the study criteria were included in this analysis. From the same cohort, 98 consecutive adult patients were selected as a disease control group, matched by age and type of epilepsy; all were attending epilepsy clinics and had recently been admitted for assessment of their epilepsy. Patients without a clear diagnosis of epilepsy were excluded, as well as those with a history of psychosis or with no clear information about personal psychiatric history.

The data collected for both cases and controls included seizure pattern, semiology, localization (if appropriate) and cause, ASM treatment and responses (including psychiatric side effects), comorbidities, presence and severity of cognitive dysfunction (categorized as none, mild, moderate, or severe based on formal testing or clinical records), brain imaging, electroencephalography (EEG)/video‐EEG telemetry, epilepsy surgery, other treatments (such as ketogenic diet and neurostimulation devices), seizure outcome, and family and personal psychiatric history.

Epilepsy causation was classified as structural, genetic, infectious, metabolic, immune, or unknown; seizures were classified as focal, generalized, or unknown^11^; focal seizures were subclassified according to localization as focal temporal or extratemporal.^12^ Focal seizures with impaired awareness, whether evolving to bilateral tonic–clonic seizures or not, may have a recollected phase without impaired awareness; we applied the term in previous usage, "aura," to such occurrences, noting that psychic auras in particular have previously been associated with postictal psychosis.^13^ Drug‐resistant epilepsy was diagnosed following established criteria.^14^ A seizure cluster or acute repetitive seizures were defined as at least one episode of multiple seizures (3 or more) occurring within a 24‐hour period in a pattern distinguishable from the baseline for that individual.^15^, ^16^

Additional patients with PIP for genetic analysis were sought from collaborators accessed via the Epi25 Consortium (www.epi-25.org). These additional cases that met the specified inclusion and exclusion criteria were included only in the polygenic risk score (PRS) analyses.

### Statistical Analysis of Clinical Data

Data were analyzed using Stata/IC V.11.1 (StataCorp, College Station, TX). Continuous variables are presented as mean ± standard deviation (SD) or median, as appropriate. Wilcoxon signed‐rank test or *t* test for continuous variables, and Pearson χ^2^ test or Fisher exact test for categorical variables, were used, as appropriate, to compare demographic and clinical data between cases and matched controls. For categorical variables that had missing data in >5% of cases, a new outcome category was added, with value "unknown" when data were missing.^17^ For the univariate analysis, the significance level was set at *p* < 0.05. Odds ratios were calculated using logistic regression to quantify associations between the occurrence of PIP and clinical factors. Univariate associations with a *p* value ≤ 0.20 were used to build a multivariate model. Variables with high collinearity (variance inflation factor > 5) were excluded from the multivariate model.

### PRS Analysis

PRSs for SCZ derived from Ripke et al^18^ were estimated in a study cohort of 2,369 people, comprising an epilepsy disease control group of 1,366 adults, another disease control group of 945 patients with SCZ (subjects meeting diagnostic criteria for the source genome‐wide association study [GWAS], but who were specifically excluded from that source GWAS),^18^ and the study case group of 58 patients with PIP (cases from NHNN and from collaborators). The epilepsy control group contains samples with available genotype data selected from the cohort of 3,288 patients who had their phenotype screened during the clinical study.

We determined the ancestry of individuals in our data by combining individual genotypes with genotypes from the 1,000 Genomes Project reference dataset,^19^ as ancestry strongly influences (PRS) analyses.^20^ Principal component analysis (PCA) on the combined data was used to detect population structure down to the level of the reference dataset. A 2‐dimensional PCA plot was used to visualize sample ancestry, and only samples from our dataset that overlapped with the European‐ancestry samples from the 1000 Genomes Project reference dataset were kept for further analysis.

Additional individual‐level quality checks were performed using PLINK 1.92. We removed all samples with <0.98 call rate for all single nucleotide polymorphisms (SNPs). Using a subset of uncorrelated SNPs (*r* ^2^ < 0.1 in a sliding window of 100 SNPs per window and shifting the window by 25 SNPs each time), we calculated heterozygosity (HET), identity by state (IBS), and gender, and removed (1) HET outliers >5 SD from the median of the whole sample, (2) one individual from each pair of closely related or duplicated individuals with >0.9 IBS across datasets, and (3) all samples where sex determined from genotype did not match with the reported gender. All SNPs with <0.95 genotype rate, <0.01 minor allele frequency, or deviation from Hardy–Weinberg equilibrium (with *p* < 1 × 10^−6^) in samples from any site were also removed.

**FIGURE 2:**
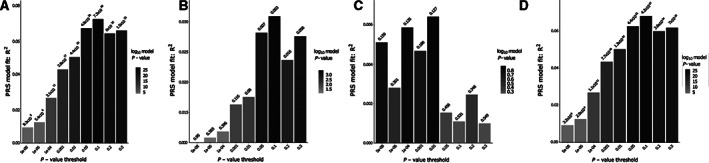
Bar plot displaying the model fit of the schizophrenia (SCZ) polygenic risk score (PRS) at different *p* value thresholds in the following models: (A) postictal psychosis (PIP) and SCZ samples versus epilepsy controls, (B) PIP versus epilepsy controls, (C) SCZ versus PIP, and (D) SCZ versus epilepsy controls.

To identify the optimal *p* value threshold (PT) for PRS prediction, we used the software PRSice v2.3.3. This program permutes the target trait values across the sample of individuals 10,000 times, and the PRS analysis is repeated on each set of permuted phenotypes. Thus, for each permutation, the “best‐fit PRS” is obtained as that most associated (higher *R* ^2^) with the target trait across the range of PTs considered.^21^ Then, as recommended in Choi and O'Reilly^21^ and as is standard practice, preserving appropriate type I error, the best predicting PT was selected for the subsequent 1‐way analysis of variance (ANOVA), and not for any PRS generation or derivation. We estimated SCZ‐PRS for the 3 cohorts (SCZ, PIP, and epilepsy controls) as follows, assuming that the biological signal for common variant risk for SCZ is the same irrespective of sample status (SCZ, PIP, or epilepsy control): we calculated PRS using PRSice in a model that included the 3 cohorts and had PIP and SCZ samples as cases and epilepsy controls as controls; in this model, the PT with the most significant *p* value found by PRSice was 10^−1^ (Fig 2).

As a complementary approach that does not force a single PT across the 3 groups being compared, we estimated PRS 3 times applying PRSice in 3 different case versus control comparisons: PIP versus epilepsy controls, SCZ versus PIP, and SCZ versus epilepsy controls. For the SCZ versus PIP model, none of the PTs was associated with a significant *p* value. For the other 2 models, the PT with the most significant *p* value was 10^−1^, and this PT was chosen also for the SCZ versus PIP model. The results from this second approach are concordant with those of the first.

Following quality control steps, we generated a SCZ‐PRS based on the overlap of the remaining (quality‐controlled) SNPs of the study groups. We used summary statistics of the GWAS for SCZ from the Psychiatric Genomics Consortium.^18^ PRS for each individual was generated using the standard application (clumping and thresholding method) implemented in the tool PRSice v2.3.3.^21^ PRSs were calculated across a range of PTs (5 × 10^−8^, 10^−6^, 10^−4^, 10^−3^, 10^−2^, 5 × 10^−2^, 10^−1^, 0.2, 0.5), and the PT with the best fit for the target trait across the range of thresholds was identified. *R* ^2^ was used to measure the variance explained by the PRS and was produced directly from PRSice.

To compare PRSs between the 3 study groups (PIP, n = 58; SCZ, n = 945, not overlapping with individuals in the source GWAS from Ripke et al^18^; and epilepsy controls, n = 1,366) for the selected (best fit) PT, ANOVA was applied. We considered the assumptions for ANOVA testing. The 3 cohorts (SCZ, PIP, and epilepsy controls) were independent. We checked that each cohort was normally distributed using the Shapiro–Wilk normality test and the homogeneity of variances using the Bartlett test. For each test, all *p* values were < 0.05.

The ANOVA model was adjusted for sex and the first 4 principal components of ancestry, which further controls for ancestry bias.^22^ We determined whether the mean difference between each pair of the groups was significant using a post hoc multiple pairwise comparison (Tukey honest significant difference) test, with significance set after Bonferroni correction to α = 0.05/3.

## Results

Of 83 patients with a history of any type of psychosis, 49 (33 male) had PIP. No difference between cases and controls was observed in duration of epilepsy (mean = 32 vs 31 years, *p* = 0.872), neurological examination findings (abnormal in 18% vs 29%, *p* = 0.381), or cognitive profile (cognitive dysfunction in 31% vs 33%, *p* = 0.761; see Table 1). Epilepsy and seizure characteristics are detailed in Table 2. The number of seizure types (median = 2 vs 2, *p* = 0.844) and the occurrence of any convulsive seizures (90% vs 87%, *p* = 0.386) were similar in both groups. Cases had a significantly higher incidence of seizure clusters (76% vs 29%, *p* < 0.001) and auras (54% vs 32%, *p* = 0.012; see Table 2).

**TABLE 1:** Comparison of Cases and Controls

General Data	Cases	Controls	*p*
Male gender, n (%)	33 (67%)	39 (40%)	0.002^a^
Mean age at last follow‐up, yr (SD)	48 (11)	44 (13)	0.051^b^
Mean age at epilepsy onset, yr (SD)	15 (12)	12 (9)	0.091^b^
Mean duration of epilepsy, yr (SD)	32 (13)	31 (16)	0.872^b^
British ethnicity, n (%)	41 (84%)	78 (80%)	0.943^c^
Abnormal neurological exam, n (%)	9 (18%)	28 (29%)	0.381^c^
Cognitive dysfunction, n (%)			
Absent	34 (69%)	62 (67%)	
Mild	12 (25%)	22 (24%)	0.761^a^
Moderate	2 (4%)	8 (9%)	
Severe	1 (2%)	1 (1%)	
Interictal background, number (%)			
Normal	35 (74%)	62 (65%)	0.267^a^
Abnormal	12 (26%)	33 (35%)	
Focal slow	7 (15%)	15 (16%)	0.890^a^
Diffuse slow	4 (9%)	7 (7%)	0.524^c^
Interictal epileptiform discharges, n (%)	32 (68%)	54 (57%)	0.197^a^
Focal interictal epileptiform discharges, n (%)			
Left temporal	6 (13%)	22 (23%)	0.136^a^
Right temporal	10 (21%)	5 (5%)	0.005^c^
Bilateral temporal	7 (15%)	9 (10%)	0.336^a^
Left anterior quadrant	4 (9%)	11 (12%)	0.404^c^
Right anterior quadrant	4 (9%)	11 (12%)	0.404^c^
Left posterior quadrant	1 (2%)	8 (8%)	0.138^c^
Right posterior quadrant	0 (0%)	4 (4%)	0.196^c^
Multifocal interictal epileptiform discharges, n (%)	3 (6%)	4 (4%)	0.446^c^
Generalized interictal epileptiform discharges, n (%)	4 (8%)	21 (22%)	0.032^c^
Neuroimaging, n (%)			
Normal	11 (22%)	35 (36%)	
Nonspecific abnormalities	4 (8%)	14 (14%)	
HS	12 (25%)	15 (16%)	0.116^c^
Other lesions	20 (41%)	25 (26%)	
Not available	2 (4%)	8 (8%)	
Epilepsy surgery, n (%)	12(25%)	17 (19%)	0.400^a^
Surgical outcome, n (%)			
Engel I–II	6 (50%)	15 (88%)	0.033^c^
Engel III–IV	6 (50%)	2 (12%)	
Vagus nerve stimulation, n (%)	7 (14%)	8 (8%)	0.248^a^
Ketogenic diet, n (%)	2 (4%)	2 (2%)	0.407^c^

^a^ χ^2^ test.

^b^ *t* test.

^c^ Fisher's exact test.

SD = standard deviation.

**TABLE 2:** Features of Epilepsy and Seizures in Cases and Controls

Epilepsy Description	Cases	Controls	*p*
Lateralization, n (%)^a^			
Left	20 (44%)	34 (38%)	
Right	18 (39%)	20 (22%)	0.017^b^
Bilateral	8 (17%)	36 (40%)	
Localization, n (%)			
Temporal	23 (47%)	31 (32%)	0.005^c^
Frontotemporal	6 (12%)	1 (1%)	
Frontal	5 (10%)	15 (15%)	
Posterior cortex	2 (4%)	5 (5%)	
Multifocal	3 (6%)	2 (2%)	
Generalized	4 (8%)	16 (16%)	
Unknown	6 (12%)	28 (29%)	
Type classification, n (%)			
Focal	45 (92%)	77 (79%)	
Generalized	4 (8%)	18 (18%)	0.130^c^
Unknown	0 (0%)	3 (3%)	
Etiology classification, n (%)			
Structural	44 (90%)	75 (77%)	0.076^c^
Genetic	5 (10%)	16 (16%)	
Unknown	0 (0%)	7 (7%)	
Seizure type, n (%)			
Focal with impaired awareness	39 (80%)	66 (69%)	0.167^b^
Focal without impaired awareness	10 (20%)	12 (13%)	0.209^b^
Focal evolving to bilateral convulsive	35 (71%)	61 (64%)	0.342^b^
Other focal seizures	2 (4%)	8 (8%)	0.280^c^
Myoclonic	4 (8%)	16 (17%)	0.123^c^
Generalized onset bilateral tonic–clonic seizures	5 (10%)	19 (20%)	0.107^c^
Absences	3 (6%)	11 (12%)	0.237^c^
Tonic/atonic	3 (6%)	7 (7%)	0.546^c^
Seizure types, n			
Median	2	2	0.844^d^
Range	1–4	1–5	
History of any convulsive seizures, n (%)	44 (90%)	83 (87%)	0.386^c^
Presence of aura, n (%)	25 (54%)	31 (32%)	0.012^b^
Seizure clusters, n (%)	37 (76%)	28 (29%)	<0.001^b^

^a^ Unknown in 3 cases.

^b^ χ^2^ test.

^c^ Fisher's exact test.

^d^ Wilcoxon rank‐sum test.

EEG showed normal background activity in most cases and controls (74% vs 65%, *p* = 0.267), with a higher incidence of right temporal interictal epileptiform discharges in cases compared to controls (21% vs 5%, *p* = 0.005; see Table 1). There was no overall difference in structural neuroimaging findings between cases and controls (normal neuroimaging in 22% vs 36%, nonspecific abnormalities in 8% vs 14%, hippocampal sclerosis in 25% vs 16%, other lesions in 41% vs 26%, *p* = 0.116; see Table 1).

All cases in our clinical series had drug‐resistant epilepsy, as did most controls, a bias related to recruitment at our tertiary referral center. There was no significant difference in the use of ASM by cases and by controls. The most frequently used ASMs in both groups were carbamazepine, clobazam, lamotrigine, levetiracetam, and valproate. No single ASM was significantly related to PIP or showed protective effects.

There was no significant difference between cases and controls in the incidence of psychiatric side effects attributed to ASMs excluding psychosis (10% vs 8%, *p* = 0.455). In the cases, drug‐related psychosis had occurred and was associated with ecstasy (n = 1), topiramate (n = 2), vigabatrin (n = 2), zonisamide (n = 1), and nitrazepam (n = 1). Drug‐induced psychotic episodes predated PIP in all instances. Psychosis was an exclusion criterion for the selected epilepsy controls.

Epilepsy surgery was performed in 12 cases (25%) and 17 controls (19%; *p* = 0.400), with no difference in mean age at surgery between the groups. Anteromesial temporal lobectomy was the most prevalent surgery type (8 cases: 5 left, 3 right; 9 controls: 5 left, 4 right). Four cases had had lesionectomy. Five controls had had lesionectomy, 1 corpus callosotomy, 1 hemispherotomy, and 1 corticectomy. Cases had a worse postsurgical outcome (Engel I–II in 50% of cases vs 88% of controls, *p* = 0.033; see Table 1). The interval between surgery and the first PIP episode ranged from 8 years before the procedure to 14 years after (mean = 0.2 years after, SD = 6.8). Other nonpharmacological treatments (vagus nerve stimulation and ketogenic diet) are documented in Table 1.

From the association analyses, the frequency of diagnosis of psychiatric disease overall was similar in cases and controls (59% vs 46%, *p* = 0.128), mood disorder and anxiety being the most prevalent diagnoses. SCZ‐like disorder and substance abuse were only present in cases. A family history of psychiatric disease was more common among cases (16% vs 4%, *p* = 0.018).

The mean age at onset of PIP was 37 years (SD = 13), and the duration of epilepsy before the first episode was long (mean = 21 years, SD = 14). The mean follow‐up after PIP was 9 years (SD = 7). The seizure types associated with an episode of PIP were convulsive in 36 (74%), nonconvulsive in 17 (35%), and unknown in 3 (6%) of the 49 cases. PIP followed a seizure cluster on at least one occasion in 74% of the patients. Treatment changes before PIP were reported as likely contributing to the psychosis by the treating physician (neurologist or neuropsychiatrist) for 11 of the 49 patients; there were 5 cases of medication change, 3 cases of noncompliance, and 3 cases of drug reduction. Hallucination occurred in 43 (88%), disorganized thinking in 41 (84%), delusion in 34 (69%), and disorganized behavior in 33 (67%) of the 49 cases.

Available for 61% of the sample, the exact duration of PIP was 1 to 2 days in 6 cases (12%), 3 to 7 days in 9 cases (18%), 8 to 15 days in 12 cases (25%), 16 to 30 days in 1 case (2%), >30 days in 2 cases (4%; one developed chronic interictal psychosis and the other experienced subsequent recurrent episodes). Treatment had to be adjusted in most cases (71%), with either introduction of antipsychotic drugs (24 cases, 56%), introduction of benzodiazepines (11 cases, 26%), or ASM adjustment (one patient was not on medication and was then started on valproate; another had his carbamazepine dose increased; and a third had zonisamide introduced). Hospitalization was required in 27 cases (66%).

A single episode of PIP was seen in 10 cases (20%). Patients commonly experienced recurrent PIP episodes (74%), whereas 5 cases (10%) developed, after resolution of PIP, psychotic symptoms unrelated to seizure frequency and were diagnosed as having chronic interictal psychosis. Analysis of PIP recurrence factors (Table 3) is limited by the small number of affected patients.

**TABLE 3:** Comparison of Cases with Single versus Recurrent Episodes of PIP

Characteristic	Recurrent Episodes, n = 39	Single Episode, n = 10	*p*
Age at epilepsy onset, yr, mean (SD)	15 (13)	18 (6)	0.044^a^
Duration of epilepsy, yr, mean (SD)	33 (13)	28 (12)	0.358^a^
Type of epilepsy, n (%)			
Focal	36 (92%)	9 (90%)	0.612^b^
Generalized	3 (8%)	1 (10%)	
Lateralization, n (%)			
Right	12 (33%)	6 (60%)	
Left	18 (50%)	2 (20%)	0.229^b^
Bilateral	6 (17%)	2 (20%)	
Electroclinical syndrome, n (%)			
Focal temporal	18 (46%)	5 (50%)	0.803^b^
Focal extratemporal	13 (33%)	4 (40%)	
Generalized	3 (8%)	1 (10%)	
Unknown	5 (13%)	0	
Presence of aura, n (%)	20 (56%)	5 (50%)	0.516^b^
Seizure clusters, n (%)	31 (80%)	6 (60%)	0.190^b^
Cognitive dysfunction, n (%)			
None	25 (64%)	9 (90%)	
Mild	11 (28%)	1 (10%)	0.543^b^
Moderate	2 (5%)	0	
Severe	1 (3%)	0	
Psychiatric side effects with antiseizure medication including psychosis, n (%)	9 (23%)	2 (20%)	0.603^b^
Personal history of psychiatric disease, n (%)	22 (56%)	7 (70%)	0.343^b^
EEG features, n (%)			
Normal background	25 (68%)	10 (100%)	0.035^c^
Interictal epileptiform discharges	24 (65%)	8 (80%)	0.307^c^
Structural neuroimaging, n (%)			
Normal	9 (23%)	2 (20%)	
Abnormal nonspecific	3 (8%)	1 (10%)	1.000^b^
Abnormal lesional, not HS	16 (41%)	4 (40%)	
Hippocampal sclerosis	9 (23%)	3 (30%)	
Not available	2 (5%)	0	
Surgical outcome, n (%)			
Engel I–II	2 (25%)	4 (100%)	0.030^b^
Engel III–IV	6 (75%)	0 (0%)	
Antiseizure medication change prior to postictal psychosis, n (%)			
No change	31 (82%)	6 (60%)	
No compliance	2 (5%)	1 (10%)	
Medication change	3 (8%)	2 (20%)	
Drug reduction	2 (5%)	2 (20%)	0.283^b^
			

^a^ Wilcoxon rank‐sum test.

^b^ Fisher's exact test.

^c^ χ^2^ test.

EEG = electroencephalographic; HS = hippocampal sclerosis; PIP = postictal psychosis; SD = standard deviation.

Univariate analyses showed significant association of PIP occurrence with male gender, right temporal epileptiform discharges on EEG, bilateral lateralization of the epileptogenic foci, presence of lesional abnormalities on neuroimaging (other than hippocampal sclerosis), history of auras, history of seizure clusters, treatment with drugs with effect on the central nervous system (non‐ASM), worse surgical outcome, and family history of psychiatric disease. Multivariate logistic regression analysis confirmed an increased risk of PIP only in association with male gender, history of auras, and seizure clusters (see Table 4 for all results from this analysis).

**TABLE 4:** Univariate and Multivariate Logistic Regression Analysis Showing Significant Association with Occurrence of PIP as Outcome Variable

Measure	Odds Ratio (univariate)	*p*	Odds Ratio (multivariate)	*p*
Gender, F	0.32	0.002	0.22	0.001
Lateralization				
Left	Reference			
Right	1.53	0.323		
Bilateral	0.38	0.043		
Right temporal epileptiform discharges on EEG	4.87	0.007		
Neuroimaging				
Normal	Reference			
Nonspecific abnormalities	0.91	0.886		
HS	2.55	0.072		
Other lesions	2.55	0.041		
History of auras	2.49	0.013	3.49	0.005
Seizure clusters	7.59	<0.001	8.57	<0.001
Treatment with drugs with effect on CNS (for comorbidities)	3.57	0.008		
Surgical outcome				
Engel I–II	Reference			
Engel III–IV	7.49	0.034		
Family history of psychiatric disease	5.17	0.022		

CNS = central nervous system; EEG = electroencephalogram; F = female; HS = hippocampal sclerosis; PIP = postictal psychosis.

**FIGURE 3:**
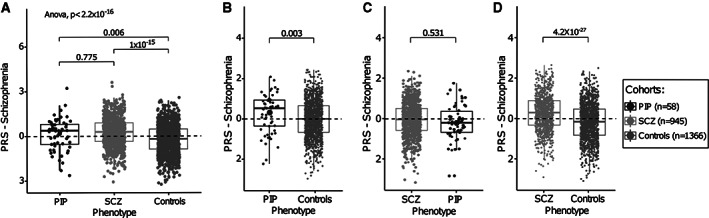
Genome‐wide schizophrenia (SCZ) polygenic risk score (PRS). (A) SCZ‐PRS estimation for postictal psychosis (PIP) and SCZ controls versus epilepsy controls (controls) for *p* value threshold = 10^−1^. (B) SCZ‐PRS estimation for PIP versus epilepsy controls. (C) SCZ‐PRS estimation for SCZ versus PIP. (D) SCZ‐PRS estimation for SCZ versus epilepsy controls.

**FIGURE 4:**
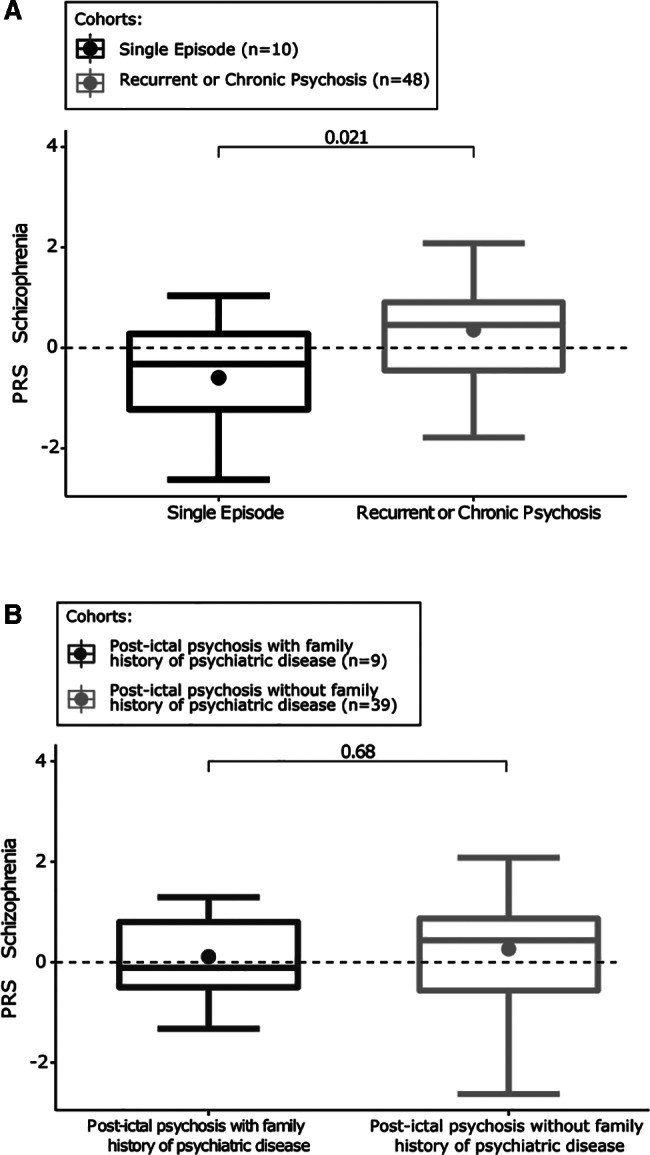
(A) Schizophrenia (SCZ) polygenic risk score (PRS) in patients with single episodes of postictal psychosis (PIP) compared to values in patients with recurrent episodes and those who went on to develop chronic psychosis. (B) SCZ‐ PRS in patients with PIP with or without a family history of psychiatric disease.

The univariate analyses suggest a possible model of genetic susceptibility to psychosis (family history), and that such psychosis requires an additional insult (in this case, seizures, typically in a cluster and on the background of chronic epilepsy) for manifestation. We hypothesized that this genetic susceptibility could be measured by a PRS for SCZ, with cases having higher PRS than in controls. Testing this hypothesis generated from the clinical data, we showed that there was a difference across the 3 groups (*R* ^2^ = 7%, *p* < 2.2 × 10^−16^, ANOVA). PRS for schizophrenia was higher in individuals with PIP than in people with epilepsy overall (unselected for a history of psychosis; *R* ^2^ = 3%, *p* = 0.006, at PT = 10^−1^, Tukey test; Fig 3). We found no significant difference in SCZ‐PRS between the PIP and SCZ groups (*R* ^2^ = 0.1%, *p* = 0.775, at PT = 10^−1^, Tukey test). As expected, SCZ‐PRS was significantly higher in people with SCZ than in individuals with epilepsy (*R* ^2^ = 7%, *p* < 1 × 10^−15^, at PT = 10^−1^, Tukey test). A complementary approach that did not force a single PT across the 3 comparisons generated concordant results. The SCZ‐PRS explained approximately 3% (*R* ^2^ = 0.03) of the total phenotypic variance in the PIP group (derived from PRSice; see Fig 2A). As exploratory analyses, we noted that SCZ‐PRS in those with recurrent episodes of PIP or chronic psychosis is higher than in those with a single episode of PIP (mean = 0.21 vs −0.42, *p* = 0.021); there was no difference in PRS in those with a family history of psychiatric disease compared with those without (mean = 0.11 vs 0.15, *p* = 0.68; Fig 4).

## Discussion

The pathophysiology of psychosis is complex and incompletely understood. Although psychosis is a central symptom in SCZ, it can be seen in a wide range of diseases and conditions, including other psychiatric diseases, such as SCZ spectrum disorders (brief psychotic, schizoaffective, delusional, and schizophreniform disorders), manic or depressive episodes in bipolar disorder, and major depressive disorder. It can occur in other conditions, including substance abuse or abrupt discontinuation, and neurological disorders such as dementia (Alzheimer disease, frontotemporal lobar degeneration), Wilson disease, Huntington disease, limbic encephalitis, and epilepsy.^23^ A multidimensional approach combining functional neuroscience and genetic susceptibility is required to produce comprehensive models to fully explain the causation.^24^ There is shared genetic liability between several psychiatric disorders, such as SCZ, major depressive disorder, bipolar disorder, and neurodevelopmental disorders, which emphasizes that psychoses do not pertain to SCZ alone, but are associated with a shared risk for mental health disorders.^25^

From multivariate analyses, we identified clinical risk factors for PIP, including male gender, seizure clustering, and the occurrence of auras. Occurrence of PIP was associated with worse outcome after epilepsy surgery. The association with a family history of psychiatric disease suggests as one possibility an underlying genetic vulnerability to psychosis.

Searching for possible causes of such an underlying vulnerability, we examined common variant burden for SCZ using a PRS approach. We show that the PRS for SCZ is higher in people with epilepsy who develop PIP than in those who do not. The actual percentage of variance explained is 3% in PIP cases, compared to 7% of trait variance explained in the independent set of people with SCZ included in this study using the PRS for SCZ generated from the largest available SCZ GWAS (see Fig 2B, C).^18^

These findings generate a model for PIP in which individuals with epilepsy with a higher PRS for SCZ (noting the lack of formal genetic correlation between SCZ and epilepsy),^26^ but without spontaneous manifestation of schizophrenia, temporarily exceed a biological threshold for manifestation of psychosis when pushed by additional factors, such as a cluster of seizures. Psychiatric and behavioral side effects of ASM are common among people with epilepsy and are particularly associated with previous psychiatric comorbidity, drug‐resistant epilepsy, and secondarily generalized seizures.^27^ More frequent use of levetiracetam was identified in patients who had developed drug‐induced psychosis, whereas carbamazepine had a protective effect; topiramate and lamotrigine were also associated with drug‐induced psychosis.^8^ We found no single drug culpable in the context of PIP, nor a protective one, which may be due to our small sample size; on the other hand, a high incidence of psychiatric side effects related to ASM overall, particularly of drug‐induced psychosis, was seen in patients with PIP.

Reportedly, 95% of PIP episodes resolve within 1 month.^28^ The mean duration of PIP is 10 days on average, varying from 1 to 63 days, with no difference comparing patients with a single episode and those with recurrent episodes.^28^ Treatment with antipsychotic drugs is associated with shortening of the episodes. Personal history of interictal psychosis, family history of psychosis, and impaired intellectual functioning have been associated with longer duration of the episodes.^28^ Interestingly, a family history of psychosis correlates with a higher PRS,^29^, ^30^ as might be expected if there is a genetic contribution to risk for psychosis. We did not detect this biological signal in the comparison between those individuals with or without a family history of psychiatric disease, but our sample was small, and family history was limited. Our PRS findings overall may be driven by a biological signal from people with recurrent episodes or who develop chronic psychosis rather than those who had had only a single episode by the time of inclusion in the study, but this inference should be considered preliminary in view of the sample size.

There are limitations to this study. These include the retrospective design, lack of detailed information in some cases, and relatively small sample size. Despite the size of our original sample, in some instances there was no information available about PIP, such that we cannot provide a figure for the incidence of PIP, but can only provide an estimate of its lower boundary (49/3,288, 1.5%). Our PRS analysis included only people of European ancestry, as the source GWAS for the generation of the SCZ‐PRS was limited to Europeans.^18^ In the PRS analysis, we did not exclude from the epilepsy control group people who had a history of psychiatric disease, but this only reduced our power to detect association. We also did not search for rare genetic variants that might increase the risk of psychosis.

The course of epilepsy in an individual may not be a linear process from diagnosis, through treatment, to seizure control. Treatments may not work, or may cause adverse effects, some of which may be severe or even life‐threatening. There may be cognitive or memory decline, episodes of status epilepticus, seizure‐related injuries, bone disease, or, as studied here, psychiatric events including PIP. Any individual with epilepsy due to any cause may have elevated risks for these or other features in epilepsy; the risks may or may not be linked to the underlying cause of the epilepsy itself. For example, an individual may have epilepsy due to an inherited stop‐gain variant in the gene *DEPDC5*, but may also have an *HLA* allele that independently increases the risk for a severe cutaneous adverse reaction to carbamazepine. Finding risk factors and the basis thereof deepens the understanding of epilepsy and of its associated manifestations in an individual, may help predict outcomes, and can influence management choices. PIP is an important neuropsychiatric manifestation in people with epilepsy. Here, we show that there are clinical factors associated with the occurrence of PIP, which act upon a higher innate risk for schizophrenia based on common genetic risk variants. More broadly, the findings suggest a testable hypothesis that the emergence of specific phenomena in a chronic condition (here, PIP) could result from the combination of genetic predisposition to those phenomena (here, elevated PRS for SCZ) and the consequences of the chronic condition itself (here, seizure clusters). If supported by empirical data, this model would add further support for the value of obtaining comprehensive individual genetic information at diagnosis for every person with epilepsy.

## Author Contributions

V.B., H.M.C., S.B., and S.M.S. contributed to conception and design of the study. V.B., L.A., S.B., G.R., H.M.C., C.L., B.W., S.C., E.B., M.G.D., C.H., F.B., Y.G.W., A.G., N.D., G.C., J.F., I.E.S., S.F.B., and S.M.S. contributed to acquisition and analysis of data. V.B., H.M.C., S.B., and S.M.S. contributed to drafting the text or preparing the figures.

## Potential Conflicts of Interest

Nothing to report.

## Data Availability

The study protocol is given in Figure 1, and the statistical analysis detailed in Patients and Methods. Data will be made available with publication. Individual level data availability is subject to specific local restrictions at each contributing center. The data can be requested by emailing the corresponding author. Data will be shared with bona fide researchers after approval of proposals with signed data access agreements as required by, and subject to, local and national regulations for each of the contributing centers.
